# Musculoskeletal Injuries in Competitive CrossFit Athletes

**DOI:** 10.1055/s-0042-1748941

**Published:** 2024-12-21

**Authors:** Kátia Sheylla Malta Purim, Adriana Zilli, Gabriela Fernandes de Almeida Leite, Gustavo Keiti Susuki, Lucas Maria S. Hapner, Matheus Antonio C. Zangari

**Affiliations:** 1 Departamento de Medicina, Universidade Positivo, Curitiba, PR, Brasil

**Keywords:** epidemiology, injuries, musculoskeletal system, physical exercise

## Abstract

**Objective** To identify the most frequent musculoskeletal injuries in CrossFit athletes who participated in a competition in 2017.

**Methods** A cross-sectional study conducted through the application of a questionnaire to adult competitors of both genders who participated in a competition in 2017.

**Results** Among the participants, 44% reported previous injuries, 67.3% of whom were men. The main types of lesions were inflammations, sprains and contusions. The most affected anatomic sites were the shoulder, spine and knee. Among the injured, 34.4% had a previous lesion at the site; 75.8% were undergoing follow-up with healthcare professionals; and all of them practiced CrossFit 5 times a week with a mean duration of the training sessions 68.2 ± 12.4 minutes and mean rest of 1.7 days a week. The duration of the training sessions was the most significant factor ( *p* = 0.002) for the occurrence of injuries.

**Conclusion** The percentage of athletes injured due to the practice of CrossFit was of 44%, with a higher incidence among men. The main type of injury was inflammation, and the most exposed anatomical sites were the shoulder, spine and knee.

## Introduction

CrossFit is a training program consisting of alternated high-intensity physical exercises. The high biomechanical and physiological demands associated with CrossFit make it paramount that healthcare professionals know its features. 1 2 3

Most injuries occur due to the improper performance of movements. 3 They can be modulated by individual factors (such as anatomical abnormalities, age, and previous injuries) and the characteristics of the training sessions (including schedule and duration). 3 4 It is noteworthy that lack of supervision increases the prevalence of injuries. 5 6 7

Regarding the rate of injuries, Summit et al. 4 reported 73,5% (3.1 injuries/thousand hours of practice), whereas Weisenthal et al. 8 identified a rate of 19% (2.4 injuries/thousand hours of training). Sprey et al. 1 showed no significant difference regarding gender, age, anthropometric data, the previous practice of other sports, the duration of the training sessions and their weekly frequency, rest time, and other concurrent physical activities. 1 However, Xavier and Martins 3 pointed out that men are 2.9 times more likely to be at risk, and that the chance of injury is 2.7 times higher among those who train for more than 1 hour. 3 This same study 3 identified overweight/obesity and weekly training frequency as risk factors. The most common injuries are contusion, strain, and tendinopathy; the most injured body parts include the shoulder, spine, and knee. 3 8 9 10 11 12

The present study aimed to identify the epidemiological profile and factors that influence the occurrence of musculoskeletal injuries in participants of a CrossFit competition held in the city of Curitiba, Southern Brazil, in 2017.

## Materials and methods

The present is a cross-sectional study approved by the institutional ethics committee (under CAAE 68129117.6.0000.0093) involving adult, regular CrossFit athletes who participated in a competition in October 2017 and signed the informed consent form. The inclusion criteria were age > 18 years, having registered at the competition, and willingness to participate in this research project. The exclusion criteria were age <below 18 years and no registration at the competition.

The sample size was calculated based on the number of participants in the competition (634, including 446 males); the final sample consisted of 333 athletes (207 men) with a 5% error.

As the research instrument, a questionnaire ( Appendix 1 ) consisting of descriptive and multiple-choice questions was developed, which was filled out and returned to the researcher during the competition. We obtained anthropometric data, as well as data pertaining to gender, features of the training sessions, the previous and current practice of other sports, the features of the CrossFit-related injuries, follow-up with a healthcare professional, and use of dietary supplements.

CrossFit-related injuries included physical complaints severe enough to require medical intervention for diagnosis and treatment; those requiring a change in the duration of the duration, intensity, or form of the training sessions for more than two weeks; or those leading to interruption of the training sessions or of other activity for more than one week. The investigators did not ask about the injury diagnosis, and they were available to clarify any doubts from the participants regarding what was considered a CrossFit-related injury.

The categorical variables were expressed as relative frequencies followed by their respective 95% confidence intervals (95%CIs), and the continuous variables, as mean and standard deviation values. The Fisher exact test for qualitative variables identified differences between strata. The quantitative variables deemed non-normal by the Shapiro-Wilk test were evaluated using the Mann-Whitney test, and the statistical analyses were performed using the Prism statistical package (GraphPad Software, San Diego, CA, United States), version 6.0.

## Results

A total of 346 athletes were willing to participate in the research; their mean age was of 28.6 ± 7.49 years. Table 1 shows the anthropometric data. Most participants (33%) had been practicing CrossFit for more than 24 months, predominantly (65%) for 60 minutes per session ( Table 2 ).

**Table 1:** Anthropometric data regarding CrossFit athletes (N = 346)

	Male	Female
**Gender (%/absolute number)**	61.9%/214	38.1%/132
**Weight (average)**	81.44 kg	61.90 kg
**Height (average)**	1.77 meters	1.64 meters

**Table 2:** Characteristics if the training sessions, rest days, and motivation to start practicing CrossFit

	Male (n = 214)	Female (n = 132)
*Time of practice (months)*		
3–6	9.8%	7.6%
7–12	20.1%	18.9%
13–18	16.4%	28%
19–24	20.6%	12.9%
> 24	33.2%	32.6%
*Number of training days per week*		
2–3	10.8%	15.9%
4–5	52.8%	47%
6–7	36.4%	36.4%
*Duration of the training sessions (minutes)*		
30	0.5%	0%
45	3.7%	1.5%
60	56.5%	79.5%
75	22.9%	11.4%
> 90	16.4%	7.6%
*Rest days*		
0–1	41.6%	48.5%
2–3	57%	47%
4–5	1.4%	3%

We observed 153 lesions (44%), 67.3% of them in males. Among the injured athletes, 33% had had previous lesions at the site. The most common injury was inflammation, and the most affected anatomical sites were the shoulder, the spine, and the knee ( Table 3 ).

**Table 3:** Frequency and distribution per age group, gender, injury type, anatomical region, and postlesion management among the injured group

*CrossFit-related injury*		
Total	153/346	
*Injuries per age group*		
18–29 years	46.8%	
30–40 years	40.5%	
41–50 years	35.4%	
*Injuries per gender*	Male	Female
	48.1%	37.9%
Type of CrossFit-related injury*		
Fracture	7.8%	6%
Contusion	19.4%	12%
Sprain	22.3%	18%
Inflammation	68.9%	78%
Dislocation	9.7%	2%
Rupture	5.8%	4%
Other	1.9%	0%
Postlesion management*		
Seeking medical assistance	70.9%	72%
Changes in training (duration, intensity, or characteristics for more than 2 weeks)	47.6%	50%
Stopping CrossFit or any other activity for more than a week	14.6%	6%
None of the above	3.9%	4%
Other	0%	2%
*Number of CrossFit-related injuries*		
1	56.3%	56%
2	29.1%	34%
3	5.8%	8%
> 3	6.8%	2%
Injured body part*		
Shoulder	56.3%	48%
Spine	25.2%	26%
Knee	19.4%	32%
Wrist	15.5%	12%
Elbow	4.9%	6%
Abdomen	3.9%	0%
Neck	2.9%	6%
Leg	2.9%	2%
Ankle	2.9%	2%
Thorax	2.9%	0%
Calf	1.9%	8%
Pelvis	1.9%	2%
Thigh	1.9%	0%
Foot	1%	0%
Hand	0%%	2%
Hip	0%%	2%
Other	2.9%	4%
*Existence of a previous lesion at the same injured site*	35%	30%

Note: *More than one alternative could be selected.

Table 4 shows the comparison between the injured and non-injured groups.

**Table 4:** Comparative analysis of CrossFit-related injuries and multiple variables

Variable	Injury		*p* -value
	*Yes (n = 153)*	*No (n = 193)*	
Female gender	32.7%	42.5%	0.075
Male gender	67.3%	57.5%	
Practice of another sport	41.4%	36.8%	0.436
Previous injury	34.4%	0.0%	0.548
Follow-up with healthcare professionals	75.8%	75.5%	1.000
Use of dietary supplements	79.7%	79.6%	1.000
Weekly frequency of the training sessions	5.0 ± 1.1	5.0 ± 1.1	0.963
Duration of the training sessions	68.2 ± 12.4	64.5 ± 10.3	0.002
Rest days	1.7 ± 0.7	1.6 ± 0.8	0.778

A total of 75.8% of athletes was followed-up regularly by healthcare professionals, mainly dieticians. Dietary supplementation was frequent, reported by 79.8% of athletes, especially women ( Table 5 ).

**Table 5:** Professional follow-up and use of supplements by CrossFit athletes

	Male	Female
***Regular follow-up with healthcare professionals***	75.2%	75.8%
Healthcare professionals (yes as previous answer)*		
Dietitian	75%	72.5%
Physiologist	11.3%	5%
Personal trainer	17.5%	14.9%
General practitioner	21.3%	13.9%
Physician nutrition specialist	16.9%	19.8%
Physical therapist	11.9%	5.9%
Other	13.1%	12.8%
***Use of dietary supplements***	76.2%	85.6%
Supplements (yes as previous answer)*		
Whey protein	90.8%	86.7%
Branched-chain amino acids (BCAAs)	50.3%	53.1%
Albumin	5.5%	5.3%
Casein	5.5%	5.3%
Creatin	41.1%	46.9%
Thermogenic compounds	17.8%	22.1%
Other	22.1%	18.6%
***Supplement prescription by healthcare professional***	***76.1%***	***77.9%***
Which healthcare professional?*		
Dietitian	50.3%	61.1%
Physician, except physician nutrition specialist	6.1%	3.5%
Physician nutrition specialist	10.4%	8%
Personal trainer	1.2%	0%
Other	0.6%	0%

Note: *More than one alternative could be selected.

## Discussion

Studies 8 report high injury rates in CrossFit due to the repetitiveness and intensity of the exercises; however, other studies 9 deny this hypothesis because of the high level of supervision and instruction during training. The present study showed an injury rate of 44%, higher than the 31% reported by Sprey et al. 1 The fact that the sample of the present study consisted only of competitive athletes explains this difference. The rates are similar to those of other sports with analogous movements (such as weightlifting and gymnastics). 2 9 In comparison to team sports, such as soccer, the percentages are lower (57% to 61.8%). 1 10 11 12 13

In the present study, the most common injuries were inflammation, sprain, and contusion, contrasting with data from the study by Xavier and Martins, 3 who observed mainly contusions. Male athletes had more injuries, which agrees with the literature. 13 On the other hand, female athletes are more careful about seeking instructions from their coach, weight overload, the frequency of training sessions, and exercise performance. 8 9

Most injuries (46.8%) occurred in the youngest athletes (18 to 29 years old), who tend to perform exercises with higher loads or repetitions due to greater impulsiveness and exhibitionism. In addition, the injury rate was indirectly proportional to age; those between 41 to 51 years old presented fewer lesions (35.7%), indicating that they may be more cautious when performing movements or they may have a better technique.

The duration of the training sessions was significant, and those who trained for longer suffered more injuries. We believe intense fatigue and lower concentration lead to erroneous movements that precipitate lesions. Consistent with the literature, 54% of the injuries affected the shoulder, 25% occurred at the spine, and 24% involved the knee. 3 8

The greater demand for dieticians (73.9%) compared to physicians (18.4%) may reveal more attention to the intake of food and dietary supplements (aiming at achieving a body with good esthetic quality) rather than comorbidities. 14 15 16 17 18 Women take more supplements, probably to perform the same training pattern as men or to change their body image. 18

These findings indicate the need to monitor, quantify and regulate the individual training load 2 16 to minimize excesses and damages to physical, psychological, and social health. 18 Sports injuries are multifactorial, and their prevention remains the best treatment. 19 20

The present study had limitations, since the information was obtained from a single competition and was dependent on the participant's memory and understanding of the concept of injury. In addition, factors such as physical and emotional stress may influence the correct completion of the questionnaire. Future epidemiological studies should focus on larger sample sizes and a high number of competitions, in order to obtain more heterogeneous and representative populational outcomes to identify other associated risk factors.

## Conclusion

In total, 44% of the athletes presented CrossFit-related injuries, including 48% of men and 38% of women. The most common injury was inflammation, and the most injured anatomical sites were the shoulder, spine, and knee. The only variable significantly associated with such injuries was the duration of the training sessions ( *p* = 0.002).

## Supplementary Material

Anexo 1 Anexo 1

## References

[1] Sprey J W Ferreira T de Lima M V Duarte A Jr Jorge P B Santili C An Epidemiological Profile of CrossFit Athletes in Brazil Orthop J Sports Med 2016 4 08 2.325967116663706E15 10.1177/2325967116663706 PMC5010098 27631016

[2] Claudino J G Gabbett T J Bourgeois F CrossFit Overview: Systematic Review and Meta-analysis Sports Med Open 2018 4 01 11 29484512 10.1186/s40798-018-0124-5 PMC5826907

[3] Xavier A A Martins A CL Lesões musculoesqueléticas em praticantes de CrossFit Rev Interdiscip Ciênc Méd 2017 1 01 11 27

[4] Summitt R J Cotton R A Kays A C Slaven E J Shoulder Injuries in Individuals Who Participate in CrossFit Training Sports Health 2016 8 06 541 546 27578854 10.1177/1941738116666073 PMC5089356

[5] Klimek C Ashbeck C Brook A J Durall C Are Injuries More Common With CrossFit Training Than Other Forms of Exercise? J Sport Rehabil 2018 27 03 295 299 28253059 10.1123/jsr.2016-0040

[6] Montalvo A M Shaefer H Rodriguez B Li T Epnere K Myer G D Retrospective Injury Epidemiology and Risk Factors for Injury in CrossFit J Sports Sci Med 2017 16 01 53 59 28344451 PMC5358031

[7] Guimarães T Carvalho M Santos W Rubini E Coelho W Crossfit, musculação e corrida: vicio, lesões e vulnerabilidade imunológica Rev Ed Física 2017 86 01 8 17

[8] Weisenthal B M Beck C A Maloney M D DeHaven K E Giordano B D Injury Rate and Patterns Among CrossFit Athletes Orthop J Sports Med 2014 2 04 2.325967114531177E15 10.1177/2325967114531177 PMC4555591 26535325

[9] Dominski F H Siqueira T C Serafim T T Andrade A Perfil de lesões em praticantes de CrossFit: revisão sistemática Fisioter Pesqui 2018 25 02 229 239

[10] Calhoon G Fry A C Injury rates and profiles of elite competitive weightlifters J Athl Train 1999 34 03 232 238 16558570 PMC1322916

[11] Nilstad A Andersen T E Bahr R Holme I Steffen K Risk factors for lower extremity injuries in elite female soccer players Am J Sports Med 2014 42 04 940 948 24500914 10.1177/0363546513518741

[12] Sousa P Rebelo A Brito J Injuries in amateur soccer players on artificial turf: a one-season prospective study Phys Ther Sport 2013 14 03 146 151 23072932 10.1016/j.ptsp.2012.05.003

[13] Moran S Booker H Staines J Williams S Rates and risk factors of injury in CrossFitTM: a prospective cohort study J Sports Med Phys Fitness 2017 57 09 1147 1153 28085123 10.23736/S0022-4707.16.06827-4

[14] Bergeron M F Nindl B C Deuster P A Consortium for Health and Military Performance and American College of Sports Medicine consensus paper on extreme conditioning programs in military personnel Curr Sports Med Rep 2011 10 06 383 389 22071400 10.1249/JSR.0b013e318237bf8a

[15] Grier T Canham-Chervak M McNulty V Jones B H Extreme conditioning programs and injury risk in a US Army Brigade Combat Team US Army Med Dep J 2013 36 47 24146241

[16] Aune K T Powers J M Injuries in an extreme conditioning program Sports Health 2017 9 01 52 58 27760844 10.1177/1941738116674895 PMC5315259

[17] Hak P T Hodzovic E Hickey B The nature and prevalence of injury during CrossFit training J Strength Cond Res 2013 10.1519/JSC.0000000000000318 24276294

[18] Rossi L Tirapegui J Insatisfação com a imagem corporal em frequentadores de academias de ginástica no Brasil Rev Bras Med Esporte 2018 24 02 162 166

[19] Barroso G C Thiele E S Lesão muscular nos atletas Rev Bras Ortop 2015 46 04 354 358 27027021 10.1016/S2255-4971(15)30245-7 PMC4799284

[20] Purim K SM Titski A CK Bento P CB Leite N Lesões desportivas e cutâneas em adeptos de corrida de rua Rev Bras Med Esporte 2014 20 04 299 303

