# Supplementary material for: Musculoskeletal Injuries in Competitive CrossFit Athletes
Source: Rev Bras Ortop (Sao Paulo). 2024 Dec 21;59(6):e976–80. doi: 10.1055/s-0042-1748941 (PMC11663056; doi:10.1055/s-0042-1748941)
Supplement: Supplementary file 1 — Anexo 1 [file 10-1055-s-0042-1748941-s1900192pt.pdf]

Anexo 1 Questionário

E-mail:	Idade:
Peso: Altura:	Gênero:

Categoria:
( ) Scaled ( ) Amador ( ) RX ( ) Master

PERGUNTAS RELACIONADAS AO CROSSFIT

01. Pratica CrossFit há quanto tempo?
( ) 3-6 meses ( ) 13-18 meses ( ) mais que 24 meses
( ) 7-12 meses ( ) 19-24 meses

02. Pratica quantas vezes na semana?
( ) 2 ( ) 3 ( ) 4 ( ) 5 ( ) 6 ( ) 7

03. Qual a duração dos treinos?
( ) 30 minutos ( ) 60 minutos ( ) mais que 90 minutos
( ) 45 minutos ( ) 75 minutos

04. Em média, quantos dias de descanso você tem na semana, sem atividade física?
( ) 0 ( ) 1 ( ) 2 ( ) 3 ( ) 4 ( ) 5

05. Porque iniciou a prática de CrossFit? (Assinalar todas alternativas compatíveis)
( ) Melhora do condicionamento físico muscular ( ) Ganho de massa muscular ( ) Definição
( ) Perda de peso médica ( ) Curiosidade ( ) Recomendação
Outros:

PERGUNTAS RELACIONADAS A OUTROS ESPORTES

06. Você praticava alguma atividade física antes de iniciar o CrossFit?
( ) Sim. Quais? Praticou por quanto tempo?
( )Não

07. Se a resposta anterior foi SIM, praticava este esporte em nível competitivo?
( ) Sim ( ) Não

08. Atualmente, pratica outra atividade física além do CrossFit?
( ) Sim. Qual? Quantos dias na semana?
( ) Não

PERGUNTAS RELACIONADAS A LESÕES DESPORTIVAS

Para melhor classificação, consideramos "lesões devido ao CrossFit" situações como:

- Qualquer queixa física severa o suficiente que necessitou ajuda médica para tratar ou diagnosticar;
- Qualquer queixa física que fez modificar o treino em duração, intensidade ou modo por mais de duas semanas;
- Qualquer queixa física que levou a interrupção dos treinos de CrossFit ou qualquer outra atividade por mais de uma semana.

09. De acordo com a definição acima, você já obteve alguma lesão devido a prática de CrossFit?
( ) Sim ( ) Não

10. Qual foi o tipo de lesão? Assinale todas as alternativas compatíveis
( ) Fratura (rompimento ou trincamento de osso)
( ) Contusão (lesão produzida por golpe ou impacto, não causa ruptura de pele)
( ) Entorse (lesão dos ligamentos devido distensão ou torção brusca)
( ) Inflamação (reação do organismo a uma infecção ou lesão dos tecidos)
( ) Luxação (ossos saem do seu ponto de articulação)
( ) Ruptura (interrupção de continuidade, divisão, corte)

11. Caso já tenha tido lesão pelo CrossFit, qual foi a sua conduta? (Assinale todas alternativas compatíveis)
( ) Procurou ajuda médica para diagnosticar ou tratar lesão
( ) Modificar o treino em duração, intensidade ou modo por mais de duas semanas
( ) Parar o CrossFit ou qualquer outra atividade por mais de uma semana
Outros:

12. Já apresentou quantas lesões devido ao CrossFit?
( ) 1 ( ) 2 ( ) 3 ( ) Mais de 3

13. Qual região do corpo foi lesionada? (Assinalar todas as alternativas compatíveis)
( ) Pescoço ( ) Ombro ( ) Cotovelo ( ) Punho ( ) Tórax ( ) Coluna ( ) Abdome
( ) Pelve ( ) Joelho ( ) Tornozelo ( ) Outros:

14. Você já apresentava lesão prévia no mesmo local lesionado durante o CrossFit?
( ) Sim ( ) Não

PERGUNTAS GERAIS

15. Você faz acompanhamento regular com algum profissional de saúde?
( ) Não ( ) Sim (ASSINALE ABAIXO TODAS AS ALTERNATIVAS COMPATÍVEIS)
( ) Nutricionista ( ) Fisiologista ( ) Personal Trainer ( ) Clínico Geral ( ) Nutrólogo
( ) Outro. Qual?

16. Você faz uso de suplemento alimentar?
( ) Não ( ) Sim

17. Se sim, qual suplemento? Assinale todas as alternativas compatíveis
( ) Whey Protein ( ) BCAA ( ) Albumina ( ) Caseína ( ) Creatina ( ) Termogênico
( ) OUTRO. QUAL?

18. A prescrição desses suplementos foi feito por algum profissional de saúde?
( ) Sim. Qual profissional?
( ) Não

19. Em média, quantos litros de água você toma por dia?
( ) Menos de 1L ( ) Entre 1L - 1,5L ( ) Entre 1,5L – 2L ( ) Mais de 2L

PERGUNTAS RELACIONADAS A SAÚDE PRÉVIA

20. Você apresenta histórico de doença renal?
( ) Não ( ) Sim. Quais?

21. Você apresenta histórico de doença cardiovascular?
( ) Não ( ) Sim. Quais? (Hipertensão, Infarto, por exemplo)

PERGUNTAS RELACIONADAS À SAÚDE FAMILIAR

22. Seus pais têm histórico de doença renal?
( ) Não ( ) Sim. Quais?

23. Seus pais apresentam histórico de doença cardiovascular?
( ) Não ( ) Sim. Quais?

 © 2022. Sociedade Brasileira de Ortopedia e Traumatologia. All rights reserved.
