# Supplementary material for: Musculoskeletal Injuries in Competitive CrossFit Athletes
Source: Rev Bras Ortop (Sao Paulo). 2024 Dec 21;59(6):e976–80. doi: 10.1055/s-0042-1748941 (PMC11663056; doi:10.1055/s-0042-1748941)
Supplement: Supplementary file 2 — Appendix 1 [file 10-1055-s-0042-1748941-s1900192en.pdf]

Appendix 1 Questionnaire

E-mail: | Age:

Weight: | Height: | Gender:

Category:
( ) Scaled ( ) Amateur: ( ) RX ( ) Master

CROSSFIT-RELATED QUESTIONS

01. How long have you been practicing CrossFit?
( ) 3–6 months ( ) 13–18 months ( ) > 24 months
( ) 7–12 months ( ) 19–24 months

02. How many times do you train per week?
( ) 2 ( ) 3 ( ) 4 ( ) 5 ( ) 6 ( ) 7

03. How long does your training session last?
( ) 30 minutes ( ) 60 minutes ( ) > 90 minutes
( ) 45 minutes ( ) 75 minutes

04. On average, how many days do you rest during the week, with no physical activity?
( ) 0 ( ) 1 ( ) 2 ( ) 3 ( ) 4 ( ) 5

05. Why did you start practicing CrossFit? (Check all alternatives that apply)
( ) Muscle fitness improvement ( ) Muscle mass gain ( ) Definition
( ) Weight loss due to medical reasons ( ) Curiosity ( ) Recommendation
Others:

QUESTIONS PERTAINING TO OTHER SPORTS

06. Did you practice any other physical activity before starting CrossFit?
( ) Yes. Please specify. ( ) For how long did?
( ) No

07. If the previous answer was YES, did you practice that sport at a competition level?
( ) Yes ( ) No

08. Do you currently engage in any other physical activity besides CrossFit?
( ) Yes. Please specify. ( ) How many days per week?
( ) No

QUESTIONS ABOUT SPORTS-RELATED INJURIES

For better classification, "CrossFit-related injuries" include:

- Any physical complaint severe enough to require medical assistance for treatment or diagnosis.
- Any physical complaint that required modifying the duration, intensity, or form of the training sessions for more than two weeks.
- Any physical complaint leading to the interruption of CrossFit training or any other activity for more than one week.

09. According to the definition stated above, have you ever suffered a CrossFit-related injury?
( ) Yes ( ) No

10. Which was the lesion type? Check all alternatives that apply
( ) Fracture (bone breaking or cracking)
( ) Contusion (injury due to blow or impact, with no skin rupture)
( ) Sprain (injury to the ligaments due to sudden strain or sprain)
( ) Inflammation (the body's reaction to infection or tissue damage)
( ) Dislocation (bones come out of their point of articulation)
( ) Rupture (continuity loss, division, section)

11. If you have ever had a CrossFit-related injury, which was its management? (Check all alternatives that apply)
( ) Seeking medical assistance for injury diagnosis or treatment
( ) Changing the duration, intensity, or form of the training sessions for more than two weeks
( ) Stopping CrossFit or any other activity for more than one week
Others:

12. How many CrossFit-related lesions have you suffered?
( ) 1 ( ) 2 ( ) 3 ( ) > 3

13. Which body region was injured? (Check all alternatives that apply)
( ) Neck ( ) Shoulder ( ) Elbow ( ) Wrist ( ) Thorax ( ) Spine ( ) Abdomen
( ) Pelvis ( ) Knee ( ) Ankle ( ) Others:

14. Have you suffered a previous lesion at the same injured site during CrossFit?
( ) Yes ( ) No

GENERAL QUESTIONS

15. Have you suffered a previous lesion at the same injured site during CrossFit?
( ) No ( ) Yes (CHECK BELOW ALL ALTERNATIVES THAT APPLY)
( ) Dietician ( ) Physiologist ( ) Personal Trainer ( ) General Practitioner ( ) Physician Nutrition Specialist
( ) Other. Please specify.

16. Do you use dietary supplements?
( ) No ( ) Yes

17. If yes, please specify. Check all alternatives that apply.
( ) Whey Protein ( ) BCAA ( ) Albumin ( ) Casein ( ) Creatin ( ) Thermogenic
( ) OTHER. PLEASE SPECIFY.

18. Were these supplements prescribed by a healthcare professional?
( ) Yes. Please specify.
( ) No

19. On average, how many liters of water do you drink per day?
( ) < 1 L ( ) 1 to 1.5 L ( ) 1.5 to 2 L ( ) > 2 L

QUESTIONS REGARDING PREVIOUS HEALTH STATUS

20. Were these supplements prescribed by a healthcare professional?
( ) No ( ) Yes. Please specify.

21. Do you have a history of cardiovascular disease?
( ) No ( ) Yes. Please specify (e.g., hypertension, heart attack).

QUESTIONS REGARDING FAMILY HEALTH

22. Do your parents have a history of renal disease?
( ) No ( ) Yes. Please specify.

23. Do your parents have a history of cardiovascular disease?
( ) No ( ) Yes. Please specify.

 © 2022. Sociedade Brasileira de Ortopedia e Traumatologia. All rights reserved.
