# Supplementary material for: Musculoskeletal Injuries in Competitive CrossFit Athletes
Source: Rev Bras Ortop (Sao Paulo). 2024 Dec 21;59(6):e976–80. doi: 10.1055/s-0042-1748941 (PMC11663056; doi:10.1055/s-0042-1748941)
Supplement: Related texts

# Research article: Lesões musculoesqueléticas em atletas de competição de *CrossFit*

**Keywords:** epidemiologia, exercício físico, lesões, sistema musculoesquelético

## Abstract

**Objetivo** Identificar as lesões musculoesqueléticas mais frequentes em atletas de *CrossFit* que participaram de uma competição em 2017.

**Métodos** Estudo transversal realizado por meio de aplicação de questionário em atletas adultos, de ambos os gêneros, participantes de uma competição em 2017.

**Resultados** Entre os participantes, 44% relataram lesões prévias, sendo 67,3% homens. Os principais tipos de lesões foram inflamação, entorse e contusão. Os locais anatômicos mais afetados foram ombro, coluna e joelho. Entre os lesionados, 34,4% apresentavam lesão prévia no local; 75,8% realizavam acompanhamento com profissional da saúde; e todos relataram praticar *CrossFit* 5 vezes por semana, com duração média dos treinos de 68,2 ± 12,4 minutos e descanso médio de 1,7 dias por semana. A duração do treino foi o fator mais significativo ( *p* = 0,002) para ocorrência de lesões.

**Conclusão** A porcentagem de atletas lesionados devido à prática de *CrossFit* foi de 44%, com maior incidência nos homens. O principal tipo de lesão foi inflamação, e os locais anatômicos mais expostos foram ombro, coluna e joelho.

## Introdução

*CrossFit* é um programa de treino em que são propostos exercícios físicos constantemente variados e de alta intensidade, e se trata, portanto, de uma modalidade de alta demanda biomecânica e fisiológica, de forma que profissionais da área de saúde devem estar cientes de suas características. 1 2 3

A exposição a lesões ocorre principalmente se os movimentos não estiverem sendo executados de maneira adequada. 3 Elas podem ser moduladas por fatores individuais (como anormalidades anatômicas, idade e lesões prévias) e/ou características do treino (como planejamento e duração). 3 4 Ressalta-se que a prevalência de lesões aumenta com a falta de supervisão. 5 6 7

Analisando as taxas de lesão, Summit et al. 4 relataram 73,5%, (3,1 lesões por mil horas de treino), ao passo que Weisenthal et al. 8 identificaram 19% (2,4 lesões por mil horas de treino). Já Sprey et al. 1 mostraram que não existe diferença significativa em relação ao gênero, à idade, aos dados antropométricos, à prática prévia de outros esportes, à duração e à frequência semanal dos treinos, ao tempo de descanso, e à prática concomitante de outros esportes. 1 Porém, Xavier e Martins 3 apontaram que homens correm 2,9 vezes mais risco, e aqueles que treinam por mais de 1 hora aumentam em 2,7 vezes a chance de desenvolver lesões. 3 Nesse mesmo estudo, 3 sobrepeso/obesidade e frequência semanal de treino foram apontados como fatores de risco. As lesões mais frequentes são contusão, distensão e tendinopatia, e as regiões corporais mais lesionadas são o ombro, a coluna e o joelho. 3 8 9 10 11 12

Este estudo visou identificar o perfil epidemiológico e os fatores que influenciam na ocorrência de lesões musculoesqueléticas em participantes de uma competição de *CrossFit* realizada em Curitiba em 2017.

## Materiais e métodos

Estudo transversal, aprovado por comitê de ética institucional (CAAE 68129117.6.0000.0093), e realizado com adultos praticantes regulares de *CrossFit* que participaram de uma competição em outubro de 2017 e assinaram o termo de consentimento livre e esclarecido. Os critérios de inclusão foram: maioridade, estar inscrito na competição, e disposição de participar deste projeto de pesquisa. e os critérios de exclusão foram menoridade e não estar inscrito na competição.

A amostra foi calculada com base no número de participantes do evento (634, sendo 446 homens), e obteve-se um tamanho amostral de 333 participantes (207 homens), com 5% de erro.

Como instrumento, elaborou-se um questionário ( Anexo 1 , material disponível on-line) com questões descritivas e de múltipla escolha, que foi preenchido e entregue ao pesquisador durante o evento. Foram obtidos dados antropométricos, e dados relativos ao gênero, às características do treino, às práticas prévia e atual de outros esportes, às características das lesões relacionadas à prática de *CrossFit* , ao acompanhamento com profissional de saúde, e ao consumo de suplementos alimentares.

**Tabela 1:** Dados antropométricos dos competidores de *CrossFit* (N = 346)

	Masculino	Feminino
**Gênero (%/número absoluto)**	61,9%/214	38,1%/132
**Peso (média)**	81,44 kg	61,90 kg
**Altura (média)**	1,77 metros	1,64 metros

Foram consideradas lesões decorrentes da prática de *CrossFit* : queixa física grave o suficiente que necessitou de intervenção médica para o diagnóstico e o tratamento; queixa física em que foi necessário modificar a duração, a intensidade, ou o modo de treino por mais de duas semanas; e queixa física que levou à interrupção dos treinos ou de outra atividade por mais de uma semana. O diagnóstico da lesão não foi questionado pelos investigadores, que estiveram presentes para esclarecer eventuais dúvidas dos participantes sobre a interpretação de o que foi considerado lesão decorrente da prática de *CrossFit* .

As variáveis categóricas foram expressas mediante frequências relativas acompanhadas pelos respectivos intervalos de confiança de 95%(ICs95%), e as variáveis contínuas, por intermédio de médias e desvios padrão. As diferenças entre estratos foram identificadas pelos testes exatos de Fisher para as variáveis qualitativas. As variáveis quantitativas, como foram reprovadas para a normalidade pelo teste de Shapiro-Wilk, foram avaliadas pelo teste de Mann-Whitney, e as análises estatísticas tiveram auxílio do pacote estatístico Prism (GraphPad Software, San Diego, CA, Estados Unidos), versão 6.0.

## Resultados

Entre os atletas, 346 se dispuseram a participar da pesquisa, e eles apresentavam idade média de 28,6 ± 7,49 anos. Na Tabela 1 estão demonstradas as medidas antropométricas. A maioria dos participantes (33%) praticava o esporte havia mais de 24 meses, com treinos predominantemente (65%) de 60 minutos ( Tabela 2 ).

**Tabela 2:** Características do treino, dias de descanso, e motivação para início da prática de *crossfit*

	Masculino (n = 214)	Feminino (n = 132)
*Tempo de prática (meses)*		
3–6	9,8%	7,6%
7–12	20,1%	18,9%
13–18	16,4%	28%
19–24	20,6%	12,9%
> 24	33,2%	32,6%
*Número de dias praticados na semana*		
2–3	10,8%	15,9%
4–5	52,8%	47%
6–7	36,4%	36,4%
*Duração dos treinos (minutos)*		
30	0,5%	0%
45	3,7%	1,5%
60	56,5%	79,5%
75	22,9%	11,4%
> 90	16,4%	7,6%
*Dias de descanso*		
0–1	41,6%	48,5%
2–3	57%	47%
4–5	1,4%	3%

Obteve-se um total de 153 de lesões (44%), sendo 67,3% em atletas do gênero masculino. Entre os lesionados, 33% apresentavam lesões prévias no local. O tipo principal de lesão foi inflamação, e os locais anatômicos mais afetados foram ombro, coluna e joelho ( Tabela 3 ).

**Tabela 3:** Frequência, distribuição por faixa etária e gênero, tipo, região anatômica e conduta pós-lesão do grupo de lesionados

*Lesão devido à prática de* CrossFit		
Total	153/346	
*Lesões por faixa etária*		
18–29 anos	46,8%	
30–40 anos	40,5%	
41–50 anos	35,4%	
*Lesão por gênero*	Masculino	Feminino
	48,1%	37,9%
*Tipo de lesão devido à prática de* CrossFit*		
Fratura	7,8%	6%
Contusão	19,4%	12%
Entorse	22,3%	18%
Inflamação	68,9%	78%
Luxação	9,7%	2%
Ruptura	5,8%	4%
Outros	1,9%	0%
Conduta após a lesão*		
Procura de ajuda médica	70,9%	72%
Modificação de treino (duração, intensidade ou modo por mais de 2 semanas)	47,6%	50%
Parar prática de *CrossFit* ou de qualquer outra atividade por mais de uma semana	14,6%	6%
Nenhuma das anteriores	3,9%	4%
Outros	0%	2%
*Quantidade de lesões devido à prática de* CrossFit		
1	56,3%	56%
2	29,1%	34%
3	5,8%	8%
> 3	6,8%	2%
Região do corpo lesionada*		
Ombro	56,3%	48%
Coluna	25,2%	26%
Joelho	19,4%	32%
Punho	15,5%	12%
Cotovelo	4,9%	6%
Abdome	3,9%	0%
Pescoço	2,9%	6%
Perna	2,9%	2%
Tornozelo	2,9%	2%
Tórax	2,9%	0%
Panturrilha	1,9%	8%
Pelve	1,9%	2%
Coxa	1,9%	0%
Pé	1%	0%
Mão	0%%	2%
Quadril	0%%	2%
Outros	2,9%	4%
*Existência de lesão prévia no mesmo local lesionado*	35%	30%

Nota: *Mais de uma alternativa podia ser assinalada.

A Tabela 4 mostra a comparação das variáveis dos grupos de lesionados e não lesionados.

**Tabela 4:** Análise comparativa entre lesões relacionadas à prática de *CrossFit* e múltiplas variáveis

Variável	Lesão		Valor de *p*
	*Sim (n = 153)*	*Não (n = 193)*	
Gênero feminino	32,7%	42,5%	0,075
Gênero masculino	67,3%	57,5%	
Prática de outro esporte	41,4%	36,8%	0,436
Lesão prévia	34,4%	0,0%	0,548
Acompanhamento por profissional de saúde	75,8%	75,5%	1,000
Uso de suplementos	79,7%	79,6%	1,000
Frequência semanal dos treinos (dias)	5,0 ± 1,1	5,0 ± 1,1	0,963
Duração do treino (minutos)	68,2 ± 12,4	64,5 ± 10,3	0,002
Dias de descanso	1,7 ± 0,7	1,6 ± 0,8	0,778

O acompanhamento regular com profissionais da saúde era realizado por 75,8% dos atletas, sendo o nutricionista o mais procurado. O uso de suplementos alimentares era comum, e 79,8% afirmaram consumi-los, com predomínio das mulheres ( Tabela 5 ).

**Tabela 5:** Acompanhamento profissional e uso de suplementos na prática de *CrossFit*

	Masculino	Feminino
***Acompanhamento regular com profissional de saúde***	75,2%	75,8%
Quais profissionais de saúde (sim, para resposta anterior)*		
Nutricionista	75%	72,5%
Fisiologista	11,3%	5%
*Personal trainer*	17,5%	14,9%
Clínico geral	21,3%	13,9%
Nutrólogo	16,9%	19,8%
Fisioterapeuta	11,9%	5,9%
Outros	13,1%	12,8%
***Uso de suplemento alimentar***	76,2%	85,6%
Quais suplementos? (sim, para a resposta anterior)*		
*Whey protein*	90,8%	86,7%
Aminoácidos de cadeia ramificada ( *branched-chain amino acids* , BCAAs, em inglês)	50,3%	53,1%
Albumina	5,5%	5,3%
Caseína	5,5%	5,3%
Creatina	41,1%	46,9%
Termogênico	17,8%	22,1%
Outros	22,1%	18,6%
***Prescrição de suplemento por profissional de saúde***	***76,1%***	***77,9%***
Qual profissional fez a prescrição?*		
Nutricionista	50,3%	61,1%
Médico (exceto nutrólogo)	6,1%	3,5%
Nutrólogo	10,4%	8%
*Personal trainer*	1,2%	0%
Outros	0,6%	0%

Nota: *Mais de uma alternativa podia ser assinalada.

## Discussão

Estudos 8 relatam altas taxas de lesão no *CrossFit* devido à repetitividade e à intensidade dos exercícios, ao passo que outros 9 negam essa hipótese por conta do elevado nível de supervisão e instrução durante a prática. Neste estudo, evidenciou-se uma taxa de lesões de 44%, resultado superior aos 31% de Sprey et al., 1 o que pode ser justificado pelo fato de a amostra ser restrita a atletas de nível competitivo. Quando comparado a outros esportes em que se realizam movimentos equivalentes (como levantamento de peso e ginástica olímpica), foram encontradas taxas similares. 2 9 No entanto, ao comparar com esportes coletivos como o futebol, as porcentagens neste último foram maiores (de 57% a 61,8%). 1 10 11 12 13

Neste estudo, as lesões mais frequentes foram inflamação, entorse e contusão, dados que contrastam com os de Xavier e Martins, 3 que relataram a contusão como principal lesão. Atletas homens apresentaram mais lesões, o que está em concordância com a literatura. 13 Já o gênero feminino se mostra mais cuidadoso quanto à procura de instrução do treinador, sobrecarga de peso, frequência de treinos, e realização de exercícios. 8 9

Nota-se que a maioria das lesões (46,8%) ocorreu entre os mais novos (18 a 29 anos de idade), provavelmente devido a maior impulsividade e exibicionismo ao realizar exercícios com mais carga ou repetições. Ademais, a taxa de lesões mostrou-se indiretamente proporcional à idade, sendo que a faixa etária entre 41 e 51 anos foi menos lesionada (35,7%), o que indica que esses atletas podem ser mais cautelosos na execução dos movimentos ou terem melhor técnica.

A duração dos treinos foi um dado significativo, que indica que os que treinam por mais tempo se lesionam mais. Supõe-se que a maior fadiga e a menor concentração levem a movimentos errôneos que precipitam lesões. Em referência à região corporal lesionada, 54% das lesões acometeram o ombro, 25%, a coluna, e 24%, o joelho, o que está em concordância com a literatura. 3 8

A maior procura por nutricionistas (73,9%) em comparação a médicos (18,4%), pode revelar maior atenção para a alimentação e suplementação (objetivando um corpo de bom aspecto estético) e menor para as suas comorbidades. 14 15 16 17 18 As mulheres consomem mais suplementos, provavelmente numa tentativa de manter o mesmo padrão de treinamento do que os homens e/ou por desejarem mudanças na imagem corporal. 18

Estes achados apontam a necessidade de monitorar, quantificar e regular a carga individual de treinamento 2 16 para minimizar exageros e danos à saúde física, psíquica e social. 18 As lesões esportivas são multifatoriais, e sua prevenção continua sendo o melhor tratamento. 19 20

O estudo apresentou limitações, uma vez que as informações foram obtidas em uma única competição, e dependeram da memória e do entendimento dos participantes sobre o conceito de lesão. Além disso, fatores como estresse físico e emocional podem ter influenciado na concentração para o correto preenchimento do questionário. Futuros estudos epidemiológicos devem conter um maior número amostral e realizar coletas em mais competições, para obter resultados mais heterogêneos e representativos da população, a fim de identificar outros fatores de risco associados.

## Conclusão

O percentual de atletas que já sofreram lesões devido à prática de *CrossFit* foi de 44% (48% dos homens e 38% das mulheres). O principal tipo de lesão foi inflamação, e os locais anatômicos mais lesionados foram ombro, coluna e joelho. Entre as variáveis analisadas, a única que se associou de forma significativa foi a duração dos treinos ( *p* = 0,002).
